# “Wing leaders” in recovery residences: staff key approaches supporting criminal legal system-involved residents receiving medication for opioid use disorder

**DOI:** 10.3389/fpubh.2025.1519469

**Published:** 2025-03-31

**Authors:** Jodie M. Dewey, James Tompkins, Amy Mericle, Dennis P. Watson

**Affiliations:** ^1^Chestnut Health Systems’ Lighthouse Institute, Chicago, IL, United States; ^2^Department of Social Work, California State University Bakersfield, Bakersfield, CA, United States; ^3^Alcohol Research Group at the Public Health Institute, Oakland, CA, United States

**Keywords:** recovery housing, substance use, medication-assisted recovery, criminal legal system, opioid use disorder, qualitative research

## Abstract

**Introduction:**

Recovery homes (also known as recovery residences or sober living homes) are an effective approach to addressing substance use disorder in the United States and have been classified into four levels of care by the National Association of Recovery Residences (NARR). These homes may be particularly successful in supporting recovery and reintegration for individuals in the criminal legal system (CLS) through increased stability and better employment outcomes, reduced recidivism, and bolstered confidence in remaining substance-free. Despite promising findings, more research across the different recovery residence levels is needed to identify the key elements that contribute to their success. This paper focuses on essential factors identified by staff working at residences which fit within NARR Level 3 for effectively supporting CLS individuals receiving medication for opioid use disorder (MOUD).

**Methods:**

As part of a larger qualitative project of recovery homes most aligned with NARR Level 3, focus groups were conducted with 19 staff members in Chicago-area recovery homes (n = 4) that serve CLS residents receiving MOUD. MAXQDA software was used to organize and analyze data.

**Results:**

As described by staff, effective support for CLS-involved residents receiving MOUD centers on two recovery goals: (1) promoting sobriety and (2) fostering personal and social stability. To encourage sobriety, recovery home staff address essential needs such as medical care, mental health support, and acquiring identity documents. These homes also waive monthly fees or what most home operators call sustainability fees (room and board). Staff also enforce strict rules and ensure active engagement with program expectations. To support personal and social stability, program content is delivered by staff with significant lived experience in both CLS and substance use.

**Discussion:**

Through programming, staff provide skills-based education to guide residents toward gradual independence aimed at destigmatizing CLS-involved residents and assisting in reintegration. However, the effectiveness of this support may be limited for those receiving MOUD as staff in this study did not actively encourage discussions about MOUD in recovery. This omission potentially reinforces stigma and hinders authentic relationships required for the social model of recovery.

## Introduction

Recovery homes (also known as recovery residences or sober living homes) are an effective and widely utilized community-based approach to addressing substance use disorders in the United States (44), providing substance-free living environments and a wide range of supportive services and network building to address and sustain long-term recovery (1). However, a paucity of research has focused on elements of recovery housing most effective to address the needs of those involved in the criminal legal system (CLS) (2). This paper highlights the key factors identified by homes categorized as Level 3 recovery homes as defined by the National Association of Recovery Residences (NARR), a national organization that defines and monitors recovery home standards. This paper investigates key ingredients staff find essential to effectively support an underserved and vulnerable population: individuals involved in the criminal legal system (CLS) who are receiving medication for opioid use disorder (MOUD).

More than 58% of those incarcerated in prisons and 63% detained in jails meet the criteria for a substance use disorder (SUD), with few receiving treatments while housed in carceral settings (3–6) and many facing barriers to treatment upon release (7–9). Social stigmas associated with SUDs and CLS-involvement limit treatment seeking and worsening health and economic disparities (10, 11). Criminal convictions often limit housing and job options (12) and strain relationships, compounding the stress of early reentry/recovery and creating instability during a time when these individuals are at the most significant risk of overdose, relapse and reincarceration (13, 14). Without recovery support services in the first 2 weeks post-release, research shows individuals with CLS involvement have a greater overdose mortality risk, with a rate 10 times higher than that of the general population (13, 15, 16). Furthermore, managing conditions of probation and parole can be daunting as restrictive court requirements sometimes interfere with recovery goals (17, 18). Recovery homes can provide the supportive environment these returning citizens need to support both their recovery and reintegration while helping residents manage court conditions (19) and ultimately break the cycle of reincarceration many CLS-involved experiences [(20), in press].

Recovery housing programs may be called different names and vary in structure and delivery of programming. However, each has a shared goal of supporting individuals as they begin and maintain recovery from substance use, obtain living-wage employment, and enter stable housing (2); this may be particularly relevant for individuals with CLS involvement (21). Recovery homes are unique from traditional treatment programs as they adopt the social model of recovery, an alternative paradigm to the clinical treatment model which focuses on formal education and relationships between patients and professionals. The social model of recovery is a peer-centered approach that values lived recovery, endorses positive role modeling, and centers the relationship among peers, staff, and the community, highlighting the importance of relationship quality to promote a level of harmony needed for a sustainable recovery (22, 23, 24, 45). However, the strategies employed by recovery homes to facilitate the social model of recovery can vary greatly. NARR has designated national standards for the 4 levels of recovery homes and defines the spectrum of recovery-oriented services Level 3 recovery homes often deliver structured programming, such as life skills classes, formal training classes, and developed recovery plans. These homes employ non-clinical staff who are trained and certified to provide direct support to residents and are supervised by a house director. Level IV homes integrate the social and medical model by delivering clinical addiction treatment while also retaining peer-support elements.

Research has shown recovery homes are particularly effective in supporting successful recovery and reintegration for CLS individuals (25). Studies examining outcomes for sober living homes (21, 26), Oxford Houses (1, 27), and clinical therapeutic communities (28) have found these social environments improve results for substance use desistance, employment achievement, mental health improvement, and recidivism reduction. However, as noted earlier, individuals with CLS involvement have unique needs, and recovery housing research has yet to identify the critical elements to serve as best practices for working with CLS residents living in recovery housing.

Further, individuals with CLS involvement and co-occurring opioid addiction face additional barriers in accessing recovery housing and other substance use treatment. Medication for opioid use disorder treatment is still highly stigmatized within the recovery community (29) despite research supporting its effectiveness (30–33). Recovery settings can reject individuals receiving MOUD and often do not recognize them as being in recovery (29, 34). Staff may hold misconceptions about MOUD which can lead to residents being encouraged to reduce or discontinue their medication [(35), unpublished]. Without uniform standards of care for individuals receiving MOUD, quality of care may differ from facility to facility (36). Even when accepting residents receiving MOUD, recovery homes can place undue pressure on residents by encouraging them to taper off MOUD too quickly, resulting in increased social and internalized stigma and greater occurrences of relapse (37). However, recovery residences that foster a non-judgmental and supportive environment for residents receiving MOUD can reduce stigmas that can undermine the formation of authentic relationships, a key element of the social model of recovery needed for sustainable recovery (38).

### Current study

To address gaps in our understanding of what may be particularly helpful for individuals involved in the CLS, receive MOUD, and reside in a recovery home, this team conducted a qualitative investigation of four Chicago-area recovery residences that serve the population of focus. Through in-depth interviews with residents and focus groups with recovery residence staff, this project investigated the challenges and best approaches of supporting CLS-involved residents who are seeking recovery from substance use. This in-depth qualitative approach (both IRB-approved and monitored), allowed researchers to center on the context-specific processes, individual decision-making, and culturally rich language participants use to discuss the phenomena under investigation (39).

## Methods

### Sites and participants

All selected recovery residences were licensed by the Substance Use Prevention & Recovery (SUPR), the only authority overseeing alcohol and substance use programs. Two of the four residences were also certified by the Illinois Association of Extended Care (IAEC), a statewide affiliate of NARR that provides training to certify recovery specialists. As a result, only two of the four homes were officially defined as NARR Level 3. While the operations of the two without this certification fall within the description of the NARR Level 3 designation, they cannot officially be labeled as such without IAEC certification. However, all four residences fit within NARR’s definition for Level 3 care as they were highly structured, employed non-clinical staff (e.g., recovery coaches, case managers) to manage residents and house rules, provided life skills programming, and supervised other house activities (NARR.org). Located in different Chicago communities and surrounding suburbs, the four recovery homes averaged 14 years in operation with a range of 4–26 years. The average bed capacity was 35 beds with a range of 7–155 beds.

**Figure 1:**
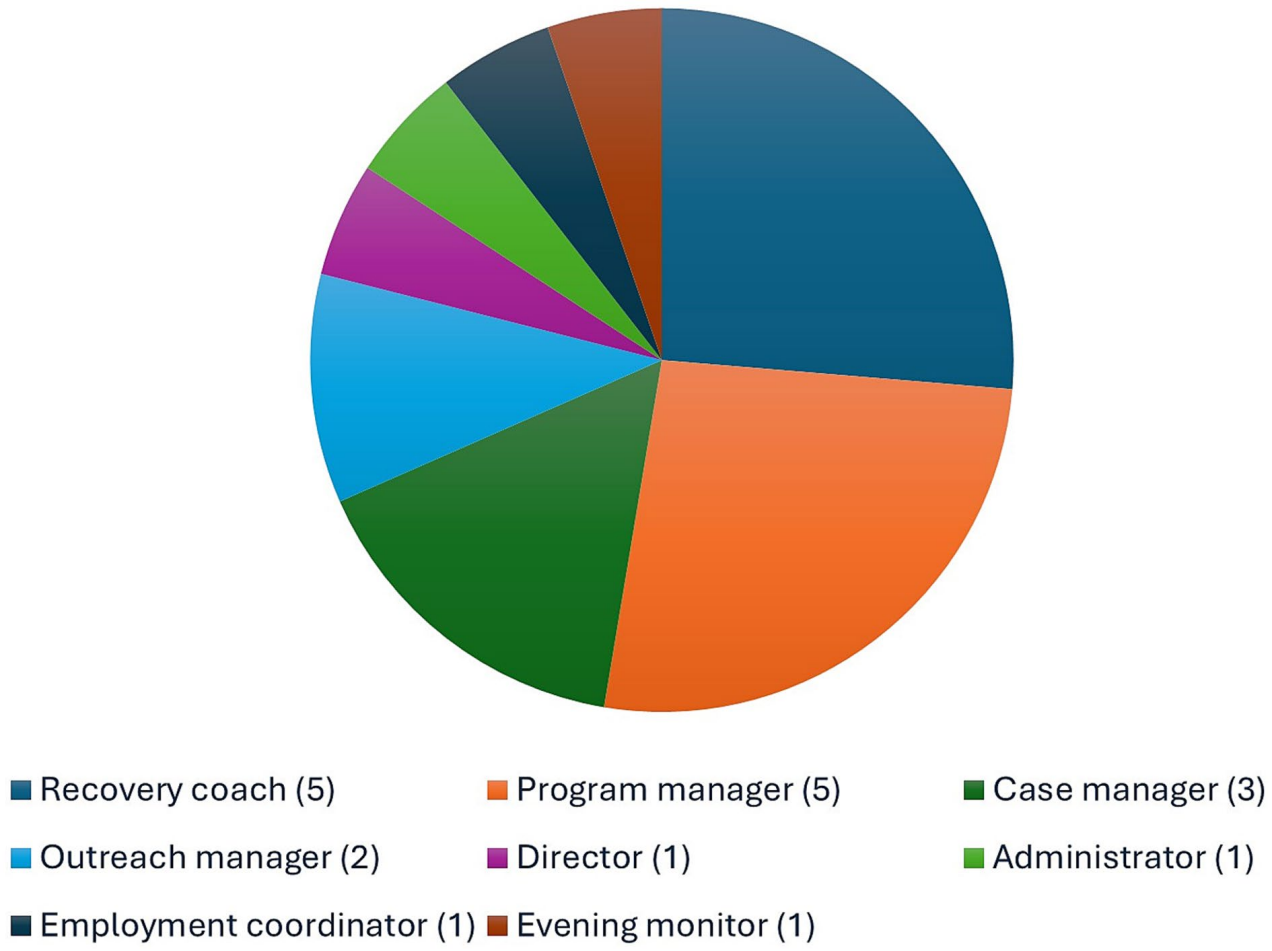
Participant roles.

This paper is solely focused on four staff focus group interviews (19 total staff participants). Researchers were initially introduced to the directors of the recovery homes included in this study through an earlier housing navigation project (19). Out of the 19 staff participants interviewed for the project, these participants held diverse roles, including director (*n* = 1), administrator (*n* = 1), employment coordinator (*n* = 1), evening monitor (*n* = 1), program manager (*n* = 5), case manager (*n* = 3), recovery coach (*n* = 5), and outreach manager (*n* = 2) (See Figure 1).

### Recruitment and data collection

Directors of each residence were contacted to discuss the project idea prior to receiving the grant supporting this work. Directors for the four participating homes provided their written commitment. Researchers were allowed on-site to introduce the project to staff and residents and, once staff who met the study’s criteria provided verbal consent, researchers arranged a time to conduct the on-site focus groups. During each focus group, researchers reintroduced the project, answered questions regarding the research, and ensured willing participants signed the informed consent.

Qualitative focus groups were conducted with staff from each of the four recovery homes. Each focus group lasted 1 h and was audio-recorded and transcribed. Names were removed during the transcription process and, to avoid identification, staff participant titles were removed before written and verbal deliverable were presented. By fostering the sharing of diverse perspectives within an organization, focus groups effectively gather insight on processes and decision-making and enable exploration of perceptions and ideas regarding context-specific topics. Participants answered 21 questions about policies and procedures for serving CLS-involved residents and what they perceived as barriers to this service. Of the 21 questions, staff were asked seven questions each about how they support clients who are CLS-involved, prescribed MOUD, and both (See Appendix). These discussions took place in private spaces at each residence, with participants reminded of the sensitivity of information shared. All participants received a $35 Visa card for their participation.

### Data analysis

MAXQDA, a qualitative analytic software (40) was used to thematically code focus group transcripts. First round coding was used to illuminate staff member experiences supporting the population of focus, drawing out explicit and implicit meanings (39, 41). Using *a priori* codes derived from the focus group questions to organize the data, researchers highlighted key points and pulled out powerful quotations (39, 42). First round coding of each focus group provided the ability to compare across cases, helping to build theoretical categories and tease out unique distinctions (41). Through an iterative process of coding and writing memos and reflections, gaps were identified and served as probes in subsequent focus groups.

Process coding was used during second round coding to cluster commonalities across processes and approaches employed by recovery residence staff to support the CLS-involved population. This analysis led to the identification of key categories representing the essential strategies and practices used by staff. Intersecting codes as identified through Code Relations Browser tool in MAXQDA elucidated dimensions of each category and allowed for the development of higher-order themes and a deeper analysis of the elements contributing to the processes under investigation (39). Preliminary data were shared with both recovery residence staff and the research team to further enhance the multidimensional facets of the data lending to the analysis presented in this paper.

## Results

Key ingredients for best serving the CLS-involved population receiving MOUD fall under two main goals of recovery as described by staff. The first goal is to help residents maintain a focused and steady path toward sobriety. The second goal is to build additional personal and social resources through CLS-focused *programming* led by staff with prior CLS and substance use involvement.

### “Getting on track”: focusing on sobriety

Whether new residents are arriving from in-patient treatment, carceral settings, or the community, sober home staff find residents need support and guidance during their initial days after arriving at the recovery residence. One recovery staff stated, “We introduce them to our recovery coaches right away, let them know who their wing leaders are” [Recovery Home-4 (RH), Ivy]. “Wing leaders are needed to help residents stay sober” (RH-2, Tom). To help residents retain focus on this critical goal, staff serve as ‘wing leaders’ by holistically addressing residents’ diverse needs to ensure sustainable success while living in the recovery home that will also set them up for success once discharged. All staff interviewed had some experience either with the CLS or recovery. Staff begin serving as a ‘wing leader’ by immediately working with each resident to identify their individual needs so they can build the physical, psychological, and spiritual foundations needed to focus on sobriety. Staff connect new residents with resources, such as medical appointments to access MOUD and enforce strict program rules while alleviating barriers that may detract from this focus.

#### Meeting essential needs: physical, psychological, and spiritual foundations for success

Due to the use of substances and/or living in carceral settings, new residents entering recovery homes frequently arrive with unmet health and social needs. Staff often must identify and address these needs before recovery-based programmatic efforts can begin.

Staff described the initial process of assessing each resident’s basic needs and ensuring access to various health services, prescribed medication, and identification documents:

We have a checklist. So, getting your physical, getting your mental health assessment, completing your substance use assessment, completing your skills assessment, your reading [skills]. Are you good at basic computer skills? Do you need support in that area? Getting their IDs, their birth certificate, Social Security card… Making sure they get those. (RH-4, Ivy)

Staff find many new residents either lost their ID or had it stolen. Acquiring identification and social security cards is required to apply for various social benefits (e.g., food assistance) and to apply for employment, something residents will eventually be prepared for. One staff member shares:

We talk to them about the value of these things and why they’re important to keep safe and keep on you. And why they need them. “You want employment? Well, they are not going to hire you unless they can prove, on your I-9, you [have] a Social Security card, an ID. (RH-4, Pam)

Those entering recovery housing often also have unmet mental health and medical needs:

You got to be dealing with mental and physical health, emotional intelligence, and [their] spiritual condition. And only when all of that is in agreement and some progress is made there, can we start making the steps to rebuild their life. (RH-3, Joe)

Aside from making appointments with medical providers, staff recognize the need to set up an appointment with a mental health care provider and why CLS-involved residents need mental health support:

[Providing] medical appointments, medication … Sometimes they need therapy sessions and stuff like that. Because a lot of them come with trauma. So, basically setting them up to have a successful discharge and go back into the community. Not to go back into the whole recidivism, into the system again,’ like a cycle. (RH-2, Jan)

To ensure residents obtain necessities, recovery residences often provide hygiene products, food, and clothing. In an effort to build a communal environment and connections across residents, homes often encourage residents share resources with newcomers. As one staff member states, “In orientation, we talk about the sense of community here. If they are hungry and they do not have something, then people on their wing will give them food or a pair of socks” (RH-4, Ivy). Another staff member iterates a similar point:

This is a community setting. This is not a one-person show and you’re going to have to pull together in order to make it happen. Mutual respect is #1. And it’s not easy to get 16 older grown men to respect each other [when they] come from different backgrounds. (RH-2, Art)

Providing essential needs for new residents and teaching them the importance of sharing with others ensures residents have what they need at each stage of their time at the recovery home.

#### Linkage to medication, including MOUD

In addition to essential needs, recovery homes in this study took additional steps to ensure new residents receiving medications, such as MOUD, had access to their prescribed medication:

We have a streamlined process and more consistent approach because one person comes in with a refill and they’re good for 60 days, and another person comes in and they have no medication for tonight. And if that [medication] is something that’s a sensitive type of thing, like schizophrenia medication, it’s important to make sure you’re consistent. I would say that’s part of it—making sure [the meds] are consistent and being managed—but [also] that their care is being managed by a professional. (RH-4, Zac)

This process of dispensing medication was similar across all recovery homes. As Level 3 recovery residences do not employ medical staff, the homes participating in this study did not employ licensed clinicians and therefore could only monitor, rather than administer medications. All four homes described similar processes for housing and monitoring medication:

Anyone that uses medication is monitored in taking their meds. They have a medical bag, but staff keep the key to the medical bag. When we distribute those meds, we give each resident their key and allow them to take their meds. Staff have to look back at their tongues to show that the meds have been swallowed. They don’t just have access to get into their meds at random will. (RH-3, Eva)

All homes held residents’ medication in a locked location and residents line up to access their locked box once in the morning and once in the evening. Residents are provided their box key, open the box, and remove their own medication. Staff do not distribute medication but ensure medications are ingested to maintain a positive recovery space free from medication diversion. While residents have privacy from other residents when accessing their lock boxes and taking their medication, anonymity regarding who is taking medication is not ensured as residents must line up together to receive medication (MOUD as well as other medications).

#### Removing sustainability fees

Staff recognize the immense pressure to regain what may have been lost through addiction and incarceration. To ensure residents can focus on their sobriety, recovery staff work to remove outside pressures and foster a distraction-free living space. One of the main pressures, especially for CLS individuals, is the pressure to work and care for others:

[Upon arriving at the recovery home] Everybody is set on, “Oh, I got to make money, or my family needs my help.” And you got to help yourself first before you try to help anybody else. Sometimes it’s disheartening because you see them trying and trying, and they’re not getting the job they want. (RH-2, Ben)

To fully focus on sobriety, residents must also be free from the pressures of self-support. The recovery homes in this project eliminated rent or what they referred to as “sustainability fees,” something uncommon in most recovery homes. The removal or delay of sustainability fees allow residents to avoid employment-related stress that could jeopardize recovery. Additionally, these programs did not require residents to forfeit any government assistance, further reducing the burden on both residents and their significant others. One staff member states, “We do not want your Link card [Illinois food assistance]. We do not want your bus card. We do not want nothing. We just want you to get on your feet.” (RH-2, Sue) Even when residents are to the point in their recovery to attain employment, recovery programs often do not request fees:

The second part is, we aid and assist our residents in acquiring employment and the only stipulation is that you save your money. Now, if that’s not helping them, I don’t know what is. Okay? And nobody else does that. We don’t ask anything from anyone. (RH-1, Tom)

#### Strict rules

Staff point to the role of strict rules and rigorous daily schedules to help residents remain focused on their sobriety and keep the pressures of community life at bay until they are ready to reintegrate. For new residents, the desire to reconnect with family and friends can undermine the single-mindedness necessary to center their sobriety, especially for those who may have spent a considerable time incarcerated:

When you get out of prison there’s a tendency to want to go out and party, celebrate. And that’s not the best long-term decision to make. So, [we] kind of put them on the right track right out of the gate. So, that’s when the case managers get involved and they say, “All right, let’s start, let’s create a list of what goals you’ve got and let’s start ticking them off.” (RH-4, Zac)

Those under community supervision may perceive court requirements as too burdensome to allow time to focus on recovery:

“I know you have legal issues, I know you have to go back to court, you have to go to probation, parole, whatever it is, your probation officer is going to come visit you.” … Stuff like that. So, just ease [the resident] back into the community. You don’t got to do everything at once. (RH-2, Jan)

To ensure adherence to program rules and for residents to remain in the recovery home environment, staff require new residents to sign an agreement:

They come into a program that has rules and regulations they have to follow in order to be stable. It’s being able to meet them halfway and tell them, “I need you to do this. If you’re not able to do this, you pretty much get your first ‘ticket,’ you’re put on probation, and if you violate that probation, then it’s termination of the program.” As soon as we meet a client, we give them a set of rules that they have to sign off on. (RH-2, Jan)

If residents cannot follow the rules, they will be required to sign a behavioral contract and placed on probation, something that will result in the loss of privileges. A co-worker continues:

If they do not follow it, they will have a behavioral contract drawn up to ensure compliance which varies depending on the participant. It’s just a warning [but] it can set them back because, when they first come in, they have a curfew of 6:00 pm and after 30 days the curfew is [changed to] 10:00 pm. (RH-2, Ben)

Staff enforce strict curfews and adherence to rigorous program schedules to keep residents busy and focused on sobriety. Residents’ days begin early with enough time to shower, dress, and get their room in order before beginning their day. For most homes, residents are not allowed to reenter their room the remainder of the day. One home explains the demanding routine:

They have meetings every day, different meetings. Anger management, an educational class, relapse prevention, basic AA [Alcoholics Anonymous] group, house issues. You got IOP [Intensive Outpatient Services], 75 hours of intensive outpatient. You got to complete 90 meetings in 90 days of self-help group, Alcoholic Anonymous, Cocaine Anonymous, faith-based, whatever you choose. (RH-2, Art)

Strict rules around movement and early curfew ensure new residents remain on-site, engaged in continual programming, and focused on their sobriety. All residences required a buddy system and prevented residents from leaving the facility during the first weeks/months unless accompanied by a fellow housemate, an individual further in their recovery who could provide immediate support to resist a return to active use.

Recovery homes also performed searches each time a resident returned to the facility, logging all property brought into the residence. In addition, staff conducted random room searches and urinalysis testing to ensure a substance-free living environment, something also required by the courts for those under community supervision. Staff perceive the strict program requirements, and the consistent routine they foster, a requirement for long-term stability:

From my experience working with this population (CLS) they don't care about the rules, they don't care about justice. So, they come into a program that has rules and regulations they got to follow in order for you to be stable. (RH-2, Jan)

### “That’s fire”: focused programming delivered by staff with lived experience

As described by staff, the key ingredient to support stability for CLS-involved residents is problem-focused programming delivered by staff who use their own lived experience with recovery, substance use, and navigating the criminal legal system All staff interviewed had some level of direct experience with both the CLS and substance use, with many having lived or currently living in the recovery home in which they were employed. This unique experience informs staff members’ approach for working with residents, mainly in the content and delivery of the programming, developing community partnerships, and helping residents redefine themselves to the community. ‘Wing leaders,’ therefore, are staff who use this personal expertise to connect with and guide residents throughout the course of their recovery.

#### Programming for CLS-involved residents

As previously mentioned, CLS residents face many pressures during reentry and recovery. Recovery residence staff stress the importance of their programs’ step by step process which allows residents to take on more responsibility as they progress:

Just the fact that to reprogram their way of thinking and the way of doing things, the way they look at life. I think one of the biggest things the fact that the people working behind the counter, our experience in the sense of life. (RH-2, Jim)

Advancing residents successfully through the process relies on an approach that can only be administered by staff with lived expertise with the CLS:

And the realness. Not all that cookie cutter, cute language, right? We’ve been hearing that there’s certain words that trigger stuff in people from our population. Like “assessments and cognitive therapy.” We hear something [that is triggering] and we automatically push back. (RH-3, Joe)

Aiming to avoid potential “triggering” language, staff with lived experience lead several in-house groups and classes especially designed to meet the needs of CLS residents in recovery:

That’s fire! [description of the classes offered by staff with lived expertise]. The morning class topics are realistic topics. We break it down to the lowest term to the point that everybody understands and everybody could relate to the topic. And Friday morning we hold a house meeting on whatever is going on right now with us right here. (RH-2, Art)

The course content, along with the unique delivery by staff who share common experiences with CLS-involved residents, is the ideal combination to best serve and support this population. Providing courses from anger management to health education, this staff member highlights “breaking it down to the lowest term” as a vital piece of the education they provide.

Programming geared specifically to the needs of CLS-involved residents is vital for successful and sustainable reintegration as residents learn to rebrand themselves and articulate a new narrative to the community as they learn new skills and change their life:

[Residents] are reconditioning themselves. I mean, it’s just like a battery in the car. It has to be reconditioned for it to continue to run. It’s not easy [maintaining sobriety]. Charge it up. (RH-3, Joe)

His coworker continues, stating:

“It’s got to be charged up and it must have some motivation in the process of being charged up to be able to give it away.” (RH-3, Flo)

Staff understand the need for continual support and motivation throughout recovery and reintegration, so residents can learn to maintain “the charge” absorbed while residing in the recovery home and successfully establish a renewed, sustainable identity. Reintroducing residents to the community is the final step to helping residents successfully reintegrate and ensuring long-term sustainability:

Connections to more opportunities as it relates to personal development and character building. [The director] got all these activities lined up to push the person to address the community of self. Because now we have to remarket ourselves. “How do I market myself as a returning citizen?” One of the things that we are big on is self-branding, right? Public speaking. We need to be able to articulate and tell our stories. (RH-3, Joe)

Part of building a new identity is connecting residents with the community. One staff member states, “We are not breathing down the neck of our clients, but we also try to maintain good behavior so the neighbors and everybody do not have complaints about us” (RH-2, Art). Another recovery home staff member states, “Eventually they [residents] are going to leave this facility and they are going to have to be able to communicate with other people outside the facility…we have to push them to do things outside of here [recovery home] and try to get them acclimated back into the community so they can be effective” (RH-4, Zac). One approach to connecting CLS-involved clients is to connect them to job programs:

[We] refer them to job programs or any type of program that supports people with a [CLS] background. We seek out an arrangement or agreement with companies and they give them the opportunity. They will overlook their convictions or their criminal record and give them a second opportunity to do anything from window washing to garbage pickup. It depends on the business. (RH-2, Jim)

One staff member discussed the importance of marketing a new identity with one of the most powerful players in many residents’ lives—their parole officer:

[We tell residents] it’s not about a time frame of getting there. It’s about getting there. Continuing the steps and the progress of what [you] have to [do]. And then you have some people say, “Well, I need you to do this and I need this to be done.” [So, they need to have a real conversation with their parole officer.] “Whoa, Whoa, Whoa. It’s so fast, you know what I’m saying? You have to work with me and at this point in time, you have power over my life. So, now I got to really sit back and be humble because you are in control. But when do I get the chance to explore who I am?” (RH-3, Eva)

Another staff member also explains why helping residents speak their story may improve the support they need for successful reentry and recovery:

If you have communication with the parole officer and the participant, collectively, all they want to know is that somebody cares. And so, if you got two people, one is the case manager who has befriended them and the other is the parole officer that they look at as that authoritative figure, and collectively they are working together, it might heighten the opportunity and the chance that [the resident] wants to do right. (RH-3, Lea)

#### Programming for residents receiving (MOUD) medications

Although all homes accepted and verbally supported residents receiving MOUD, the homes varied in how they supported those receiving MOUD. Only one staff member discussed the inclusion of a general medication management course in their homes’ programming, stating:

We have a dual diagnosis class to teach them how to properly take the medication and what side effects of these medications can occur. Letting residents know to drink plenty of water with any medication that you take. You have to be very careful when it comes to psychotropic medication. (RH-3, Eva)

Although the course did not specifically address MOUD in their programming, staff had a clear response on how they would handle any resident who had a problem with someone on MOUD, stating:

And if you are uncomfortable with somebody’s medication, then [we are] very good at referencing you out. You cannot come and change the rules of [our recovery home]. It’s a place for comfort, nondiscriminatory against your medication. However, if that’s uncomfortable for you, we can refer you somewhere else. (RH-3, Lea)

This home was the only home that provided visible educational material on overdose prevention. When visiting the home, the lead researcher observed the material on a small table in the dining room and the director of the home discussed the importance of keeping residents safe and the ability to help each other if needed.

As previously mentioned, although residents receiving MOUD were accepted at all participating recovery homes, support for these residents was not clearly defined. When asked how they would handle a resident who began to talk about receiving MOUD as a part of their recovery during a class or meeting at the residence, one staff member provided this response:

It’s touchy. As facilitators, we don’t ask these questions [whether someone is on MOUD]. But if you are bringing it up, anywhere from sexuality to medication, we will entertain it and respect it and hear you out. But we don’t bring it out of them. (RH-2, Art)

The reasons for the lack of supportive programming may be due to confusion regarding whether MOUD should be included in recovery house programming. Staff at two recovery homes preferred, no one could tell if residents were receiving MOUD:

Our policy is that as long as nobody in the building can tell that you’re on any type of MOUD, the treatment, then we’re fine with it. So, therefore, if you walk through the building, you probably would have no idea which of the guys that we have currently are on any type of treatment like that. Essentially, they’re just like anybody else, from our perspective. And if they are on too high of a dosage, then maybe they need to taper down a little bit. (RH-4, Zac)

Later Zac describes ‘nodding’ off [falling asleep, a stigmatized term sometimes used to infer being prescribed too high a dose (See 46)] or sleeping during programming as evidence that one’s medication is too high. Echoing a similar perspective, one staff member shared their perspective on methadone, stating: “For the methadone, that’s the expectation: for them to decrease [the dosage] as they are here. And for the Suboxone, there are people that just want to get off of it.” (RH-2, Ben) The main concern participants in focus groups expressed with residents’ medication is that they will be unable to participate in programming or create emergency situations in the residence. Although admitting she did not know how many milligrams was too high, Lea explains when one’s medication dosage is problematic:

I could say from just experience, it is very important to know a dosage. Some psychotropic medication, the dosage will cause you to be in Zombieland. So, it's important to be aware, to minimize them of what they take in the dosage that they're taking due to the fact that it can cause different behavior patterns in the home. It could cause problems if they have too much, and it can cause [problems] if they are not taking it. (RH-3, Lea)

Staff at another home elaborates and clarifies the concern as it applies specifically to MOUD with one staff member stating, “The only policy and procedure we have, because we are not doctors, so we do not have the right to determine anybody’s dosage, you just cannot be nodding. You cannot look like you are high [when on MOUD] (RH-1, Bob). His colleague echoes this point:

As long as you’re able to participate and follow direct orders and don’t look like you are nodding, not sleeping. And we tell them [resident] to go and see their doctor [about] their medication. Because obviously something’s going on there with their medication that is not keeping them alert. (RH-1, Sue)

Another staff member from a different recovery home speaks about the potential pressure to reduce one’s MOUD dosage:

Since most people don't start their dosage while they're here, they probably have already been on it prior to arrival. The doctors probably already had a chance to taper down or find the right dosage, because the doctor also does not want them to be nodding off like that. Because that means you're overprescribed. 90% of the people, they've already found a good dosage prior to arrival. So, oftentimes they'll stay on that. Maybe they gradually taper down. I wouldn’t say it’s a topic that comes up very often. And oftentimes it’s caught by the person themselves or the doctor. And the person wants to stay in recovery, ostensibly the person wants to stay in recovery, so if they feel like they’re getting a high out of it, maybe they’re getting a nodding off feeling, they might taper down themselves. (RH-4, Zac)

## Discussion

Recovery residences are an evidence-based approach to meet the multiple recovery and reintegration challenges experienced by CLS-involved individuals recovering from substance use disorder (27, 47–49). The findings presented in this paper fill two important gaps in the literature. This study first examines the key elements identified by staff as essential for effectively supporting criminal justice system (CJS)-involved residents receiving MOUD (22). Secondly, it provides an in-depth analysis of the role recovery staff with lived experience play in adapting recovery home programs for justice-impacted individuals, a population often characterized by mistrust and marginalization, particularly when it comes to accessing recovery housing (29). The qualitative findings highlight two crucial factors for successful recovery: (1) a clear emphasis on minimizing all barriers that detract from sobriety and (2) the establishment of personal and social stability. Additionally, the data suggests that recovery staff with lived experience may be the critical element needed to root CLS-involved residents in recovery housing, an evidence-based intervention with considerable benefits for this population (1, 21, 25–28). ‘Wing leaders’ use their expertise to build trust, address individual needs, confront particular personal challenges that detract from successful recovery, and deliver powerful programming that can reach this vulnerable population.

New residents are immediately assigned a “wing leader” to help residents “get on track.” As a self-designation, staff use this term to describe how they use their own personal experiences with the CLS and recovery to connect, build trust, and guide new residents through the recovery process. This lived experience is one of the key ingredients that shape other crucial elements of recovery housing. Recognizing the pressure CLS involved residents feel to regain what they lost through incarceration and substance use, staff ease residents’ initial fears and stress related to early reentry and recovery (13, 14). Residents are provided with essential needs staff believe are required for residents to maintain their focus on sobriety. Staff help residents acquire necessary identification documents, schedule medical and mental health appointments, and obtain prescribed medications, including MOUD, essential efforts that improve positive outcomes previously noted by those researching recovery residences (1, 21, 26, 27). To ensure residents can center their sobriety and fully participate in the rigorous recovery home programming (8), recovery homes in this study do not impose rent or sustainability fees. By removing fees, homes find that residents can delay the pressure they feel to support themselves and family, and can delay the requirement to work placed upon them by the CLS (18). Recovery homes provided necessities including toiletries and food and encouraged residents to share resources with each other, an early step designed to establish bonds among the residents, a key component of the social model of recovery. By holding residents accountable and nurturing relationships among residents, staff foster transformative connections helping residents receive the support needed during initial stages of recovery while promoting the positive role modeling needed for steadiness often required for long-term recovery (23). This dynamic has been highlighted in research on homophily within recovery home settings, which suggests that shared experiences and relationships among residents contribute to enhanced recovery outcomes (22, 24).

To further assist residents in maintaining focus on their sobriety, recovery home staff serve as the primary representatives of the program, tasked with communicating the rules and requirements of the residence. Their role is integral in ensuring residents understand and adhere to the expectations set forth to support their recovery journey. Through required rigorous daily programming, as well as strict rules around curfew and use of a buddy system, staff keep residents accountable to the program and to each other. With a full schedule of program-related events, residents lack the time to do anything but focus on their recovery. Through this early intervention, staff orient new residents at a time when they are at the highest risk of relapse, overdose and reincarceration (13, 15, 16). Even when residents may not fully understand or appreciate the rules, they place their trust in staff members, recognizing that the staff’s personal experiences uniquely position them to offer guidance. Having come from similar backgrounds and having navigated the same challenges, staff are seen as the most capable individuals to support residents on their journey. In this context, the term “wing leader” is particularly fitting, as it conveys someone who is steadfastly in your corner and offers unwavering support.

Staff incorporate their lived experience into required meetings and courses to model and promote personal and social stability for CLS-involved residents. Staff provide courses on life skills, various health topics, anger management, self-care, vocational training, Alcoholics/Narcotics Anonymous, and navigating community supervision requirements. Staff attribute the success of their courses and the ability to empower residents to their lived expertise. They present their unique approach as an amalgam of skill-based programming with the ability to keep it “real.” Avoiding what they feel is “cookie cutter” programming and potentially triggering language often associated with clinical approaches of existing programs, staff find their unique presentation of the material helps residents both trust the information and absorb its importance. Furthermore, staff engage residents through a slow and incremental course sequencing, something staff find vital for sustainable recovery and stable independence. Therefore, types of programming and delivery styles can be a key feature of these structured, staff-led recovery residences, further promoting the stability already reported in existing recovery research (1, 2, 26, 43). In contrast to other studies, the data presented here offers a comprehensive analysis of how recovery home staff effectively implement recovery programs. It underscores the critical role of lived experience in engaging and retaining new residents, particularly in supporting the challenging efforts required for both recovery and successful reentry. Given the important role that staff with lived experience play in residents’ recovery journeys, it is important to investigate how they are able to maintain their own recovery and the potential role that recovery homes can play in supporting their staff members in recovery.

Despite the impressive course line-up to support recovery, the homes participating in this study did not provide courses specific to MOUD. In fact, they differed immensely on whether they allowed residents receiving MOUD to speak to the role MOUDs play in their recovery. Without a consistent culture of MOUD acceptance, misconceptions about MOUD lead staff to encourage residents to lower their subtherapeutic dose or discontinue treatment, potentially further stigmatizing those prescribed this life-saving medication [(29, 35), unpublished].

The decisive step for staff to encourage social stability and ensure sustainable long-term recovery is connecting CLS residents with the broader community. Staff dedicate time to establishing positive partnerships with local organizations, thus facilitating access to essential services as residents transition into community life. Recognizing that stigma related to substance use and criminal histories poses significant barriers, staff collaborate with employment services to address employer concerns regarding hiring individuals with criminal backgrounds. This proactive approach builds trust and confidence and strengthens the ties between staff and residents, essential for the success of the social model of recovery (23). This approach also fosters opportunities for meaningful employment in organizations willing to embrace them, potentially reducing existing stigmas faced by this population, improving residents’ confidence, and reducing economic disparities associated with lack of employment (10). Staff also empower residents under community supervision to develop positive relationships with their probation and parole officers. Establishing communication with officers may improve successful completion of court conditions and reduce the potential conflict of court requirements with recovery goals (17, 18). Moreover, opportunities for residents to share their successful recovery journey with supervising officers may alleviate stigmas potentially held by CLS partners and lead to improved mutual understanding, potentially breaking what some define as the revolving door of the justice system.

Staff in the recovery homes studied, which fit best in the rubric of the NARR Level 3, utilize what one participant described as the “social suggestive model of recovery.” The data highlights how this enhanced model supports residents’ recovery journey through skill-building programs led by staff with lived expertise. As essential “wing leaders” guiding and supporting residents, we recognize that it is not only the resources provided by recovery homes that matter, but how these resources are delivered. This approach offers a promising avenue for investigation, potentially leading to long-term, sustainable recovery success. By fostering deeper connections between residents, staff, peers, and the wider community, staff employed at NARR Level 3 homes slowly and incrementally help residents build stability and a sustainable future. This model appears to be highly effective for CLS-involved clients, providing not only the support needed to remain in recovery but helping CLS residents build connections with others and establish the new identities required to successfully reintegrate. It is important to note, however, that the effectiveness of this approach may be hindered if residents are unable to authentically discuss MOUD, as this could perpetuate stigma surrounding this life-saving treatment and those who need it. As Gallardo et al. (38) illustrate, without a non-judgmental recovery environment residents can internalize stigma. If recovery homes hope to empower residents, staff need to acknowledge all forms of stigma, as residents may struggle to fully engage in the program if they cannot openly discuss their recovery objectives.

## Data Availability

The original contributions presented in the study are included in the article/Supplementary material, further inquiries can be directed to the corresponding author.
