# Supplementary material for: “Wing leaders” in recovery residences: staff key approaches supporting criminal legal system-involved residents receiving medication for opioid use disorder
Source: Front Public Health. 2025 Mar 31;13:1519469. doi: 10.3389/fpubh.2025.1519469 (PMC11994662; doi:10.3389/fpubh.2025.1519469)
Supplement: Supplementary file 1 [file Data_Sheet_1.docx]

**Staff Focus Group Question Guide**

**Good morning/afternoon. I want to thank all of you for your willingness to participate in this focus group.**

- [Introduce yourself by stating your name, current role, years at your organization, and providing a brief description of your responsibilities.]

I would like to kindly remind you to not share other people’s names or identities, or their responses from the focus group so that we can maintain the privacy and trust of the participants in this project. As a reminder, what you share is up to you. If any question makes you uncomfortable, you may choose not to answer it.

I do want to hear from all of you throughout our meeting today so just let me know when you have input, please! First, could someone start with a general overview of your organization and what it does? [Probe and start bringing in other participants who are more reticent to join in the conversation.

I would like to begin our conversation by speaking with you about your experience placing and housing clients who are involved in the criminal legal system.

- CLS-Involvement
- Please describe the policies and procedures for housing individuals who have been involved in the criminal and legal system.
  - What informs these policies? [Probe: Model, standards, etc.]
  - Has anything shaped updates or changes to these policies?
- What are the biggest challenges to implementing these policies and procedures when housing this population?
- Describe a typical process for placing these clients at your organization.
- Describe a typical process for housing these clients at your organization. [Probe: Identify any unique house rules, legal support, needs and how they are addressed, etc.]
- What are the biggest challenges to placing and housing this population?
- Can a few of you share anything you feel facilitates your ability to effectively work with this population? [Use this and the next question to draw out any participants who have not contributed.]
- Is there anything your organization could implement or change to support this population more effectively?

Thank you. Now let’s move to housing clients who are using medication for opioid use disorder or MOUD clients.

MOUD

- Please describe the policies and procedures for housing clients using medication for opioid use disorder.
  - What informs these policies? [Probe: Model, standards, etc.]
  - Has anything shaped updates or changes to these policies?
- What are the biggest challenges to implementing these policies and procedures when housing clients using medication for opioid use disorder?
- Describe a typical process for placing these clients at your organization.
- Describe a typical process for housing these clients at your organization. [Probe: medication limits and dispensing, rules around participation, etc.]
- What are the biggest challenges to placing and housing this population?
- Can some of you share anything that you feel facilitates your ability to effectively work with these clients?
- Is there anything your organization could implement or change to support this population more effectively?

Thank you. I appreciate your input. Now let’s talk about your experiences serving those clients who are CLS-involved and receiving MOUD.

Serving CLS-Involved Clients Receiving MOUD

- Describe how your organization came to house/support this client group.
- In what ways is your organization most effective in supporting this particular client group? [Probe: essential/counterproductive elements; work to bring in all participants]
- What challenges have you personally experienced or witnessed working with these clients? [Probe: peer stigma, program requirements, etc.]
  - How have you and your organization responded to those challenges?
- How have you, as a staff member, had to change or shift the way you approach your work when supporting these clients?
- How do you think these clients perceive their experiences at your site?
- Is there anything your organization could implement or change to support this population more effectively? [Probe: organization v. staff-level changes]
